# Crystal structures of *N*-[(4-phenyl­thia­zol-2-yl)carbamo­thio­yl]benzamide and *N*-{[4-(4-bromo­phen­yl)thia­zol-2-yl]carbamo­thio­yl}benzamide from synchrotron X-ray diffraction

**DOI:** 10.1107/S2056989016013396

**Published:** 2016-08-26

**Authors:** Ekaterina S. Gantimurova, Alexander S. Bunev, Kristina Yu. Talina, Gennady I. Ostapenko, Pavel V. Dorovatovskii, Nikolai N. Lobanov, Victor N. Khrustalev

**Affiliations:** a Laboratory of Functional Heterocyclic Compounds, Togliatti State University, 14 Belorusskaya St., Togliatti 445020, Russian Federation; b National Research Centre "Kurchatov Institute", 1 Acad. Kurchatov Sq., Moscow 123182, Russian Federation; c Inorganic Chemistry Department, Peoples’ Friendship University of Russia, 6 Miklukho-Maklay St., Moscow 117198, Russian Federation; d X-Ray Structural Centre, A.N. Nesmeyanov Institute of Organoelement Compounds, Russian Academy of Sciences, 28 Vavilov St., B-334, Moscow 119991, Russian Federation

**Keywords:** crystal structure, thio­urea, thia­zoles, synchrotron, hydrogen bonds

## Abstract

The title compounds, C_17_H_13_N_3_OS_2_, (I), and C_17_H_12_BrN_3_OS_2_, (II), are potential active pharmaceutical ingredients. Compound (I) comprises two almost planar fragments. The first is the central (carbamo­thio­yl)amide (r.m.s. deviation = 0.038 Å), and the second consists of the thia­zole and two phenyl rings (r.m.s. deviation = 0.053 Å). The dihedral angle between these planes is 15.17 (5)°. Unlike (I), compound (II) comprises three almost planar fragments. The first is the central *N*-(thia­zol-2-ylcarbamo­thio­yl)amide (r.m.s. deviation = 0.084 Å), and the two others comprise the bromo­phenyl and phenyl substituents, respectively. The dihedral angles between the central and two terminal planar fragments are 21.58 (7) and 17.90 (9)°, respectively. Both (I) and (II) feature an intra­molecular N—H⋯O hydrogen bond, which closes an *S*(6) ring. In the crystal of (I), mol­ecules form hydrogen-bonded layers parallel to (100) mediated by N—H⋯S and C—H⋯O hydrogen bonds. In the crystal of (II), mol­ecules form a three-dimensional framework mediated by N—H⋯Br and C—H⋯O hydrogen bonds, as well as secondary S⋯Br [3.3507 (11) Å] and S⋯S [3.4343 (14) Å] inter­actions.

## Abstract 2

The crystal structures of two new thio­urea derivatives – potential active pharmaceutical ingredients (APIs) – were studied by synchrotron X-ray diffraction.

## Chemical context   

Thio­ureas are the subject of significant inter­est owing to their biological properties as fungicides, herbicides (Walpole *et al.*, 1998 ▸) and rodenticides (Sarkis & Faisal, 1985 ▸). It is also well-known that thio­urea derivatives and their metal complexes exhibit analgesic (El-Serwy *et al.*, 2015 ▸), anti-inflammatory (Lin *et al.*, 2013 ▸), anti­microbial (Stefanska *et al.*, 2016 ▸) and anti­cancer (Rauf *et al.*, 2015 ▸) activities. Moreover, thio­urea derivatives are valuable building blocks for the synthesis of amides, guanidines and a variety of heterocycles (*e.g*. Kidwai *et al.*, 2001 ▸; Du & Curran, 2003 ▸). Recently, thio­urea derivatives were found to have use in organocatalysis (*e.g.* Connon, 2006 ▸; McCooey & Connon, 2005 ▸; Schreiner, 2003 ▸; Taylor & Jacobsen, 2006 ▸). For these reasons, a number of procedures have been reported for the synthesis of thio­ureas.

In this paper we report a synthetic approach for the preparation of the new thio­urea derivatives (I) and (II) containing thia­zole fragments, and their structural characterization by synchrotron single-crystal X-ray diffraction.
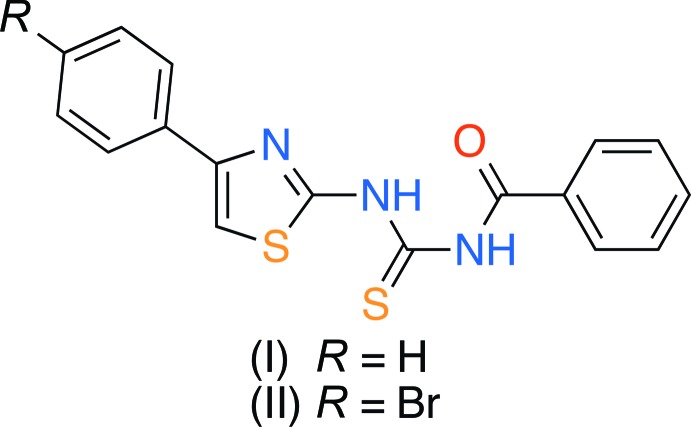


## Structural commentary   

**Figure 1:**
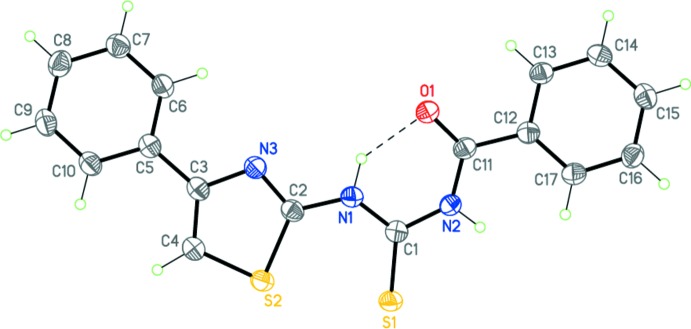
The mol­ecular structure of (I). Displacement ellipsoids are shown at the 50% probability level. The dashed line indicates the intra­molecular hydrogen bond. H atoms are presented as small spheres of arbitrary radius.

Compound (I), C_17_H_13_N_3_OS_2_, comprises two almost planar fragments. The first is the central (carbamo­thio­yl)amide grouping (r.m.s. deviation = 0.038 Å), and the second consists of the thia­zole and two phenyl rings (r.m.s. deviation = 0.053 Å) (Fig. 1 ▸). The dihedral angle between these planes is 15.17 (5)°.

**Figure 2:**
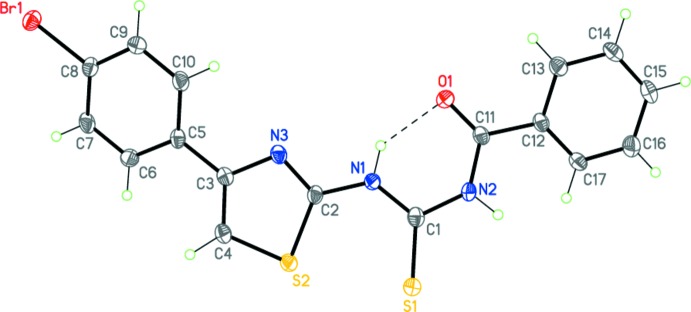
The mol­ecular structure of (II). Displacement ellipsoids are shown at the 50% probability level. The dashed line indicates the intra­molecular hydrogen bond. H atoms are presented as small spheres of arbitrary radius.

Unlike (I), compound (II), C_17_H_12_N_3_OS_2_Br, comprises three almost planar fragments: the first is the central *N*-(thia­zol-2-ylcarbamo­thio­yl)amide (r.m.s. deviation = 0.084 Å), and the two others comprise the bromo­phenyl and phenyl substituents, respectively (Fig. 2 ▸). The dihedral angles between the central and two terminal fragments are 21.58 (7) and 17.90 (9)°, respectively.

The planarity of the fragments found in (I) and (II) is determined by the present of bond conjugation within each of them as well as the intra­molecular N1—H1⋯O1 hydrogen bond (Tables 1 ▸ and 2 ▸, Figs. 1 ▸ and 2 ▸). The different mol­ecular conformations observed for (I) and (II) may apparently be explained by the various systems of inter­molecular inter­actions present in the crystals (see the *Supra­molecular features* section below).

The bond-length and angle distribution within mol­ecules (I) and (II) are almost identical and in good agreement with those observed in related compounds (Singh *et al.*, 2012 ▸, 2013 ▸). The values for the C—S—C angle in (I) [88.06 (8)°] and (II) [87.75 (14)°] are also very close to those in previously reported analogous structures [87.62 (7)–88.11 (8)°] (Yunus *et al.*, 2008 ▸; Saeed *et al.*, 2010 ▸).

## Supra­molecular features   

Although the similarity of the mol­ecular geometries and types of intra­molecular hydrogen bonds might lead to similar packing motifs, this is not found in the case of (I) and (II). The inter­molecular inter­actions, namely, N—H⋯*X* (*X* = S, Br) and C—H⋯O hydrogen bonding and the secondary S⋯S and S⋯Br inter­actions, combine in a different way, give rise to distinct packing motifs.

**Figure 3:**
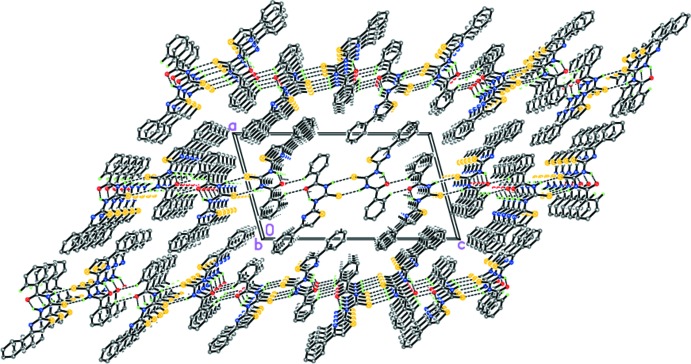
The crystal structure of (I) illustrating the hydrogen-bonded layers parallel to (100). Dashed lines indicate the intra­molecular N—H⋯O and inter­molecular N—H⋯S and C—H⋯O hydrogen bonds.

In (I), the crystal packing consists of hydrogen-bonded layers parallel to (100), in which the mol­ecules are linked to each other by N2—H2⋯S1^i^ and C13—H13⋯O1^ii^ hydrogen bonds [Table 1 ▸, Fig. 3 ▸; symmetry codes: (i) −*x* + 1, −*y* + 1, −*z* + 1; (ii) −*x* + 1, *y* − 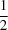, −*z* + 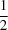]. No secondary S⋯S inter­molecular inter­actions were observed in (I).

**Figure 4:**
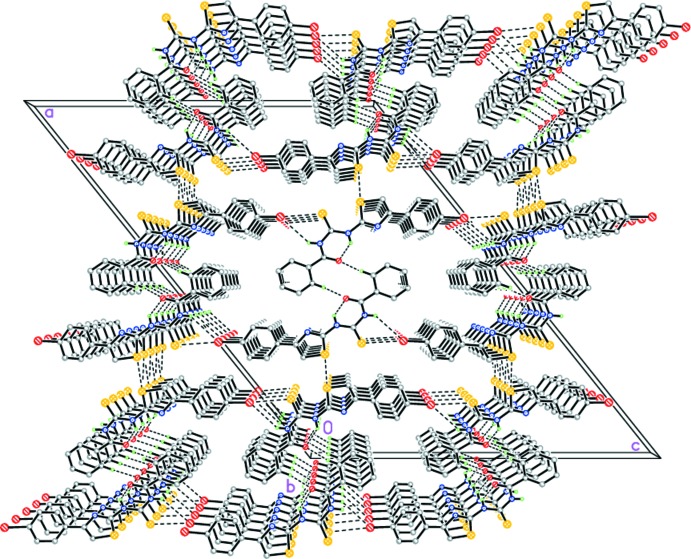
The crystal structure of (II). Dashed lines indicate the intra­molecular N—H⋯O and inter­molecular N—H⋯Br and C—H⋯O hydrogen bonds, as well as secondary inter­molecular S⋯S and S⋯Br inter­actions.

The situation in the case of (II) is quite different. The mol­ecules of (II) form a three-dimensional framework mediated by the N2—H2⋯Br1^i^ and C13—H13⋯O1^ii^ hydrogen bonds (Table 2 ▸, Fig. 4 ▸) as well as the secondary S1⋯Br1^iii^ [3.3507 (11) Å] and S2⋯S2^iv^ [3.4343 (14) Å] inter­actions [symmetry codes: (i) *x*, −*y* + 1, *z* − 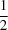; (ii) −*x* + 1, −*y*, −*z* + 1; (iii) *x*, −*y* + 1, −*z* + 1; (iv) −*x* + 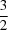, *y* + 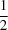, −*z* + 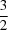; Fig. 4 ▸]. It should be pointed out that the secondary inter­molecular S⋯Br and S⋯S inter­actions in (II) are significantly stronger than the inter­molecular hydrogen bonds and, consequently, structure-forming.

## Synthesis and crystallization   

**Figure 5:**
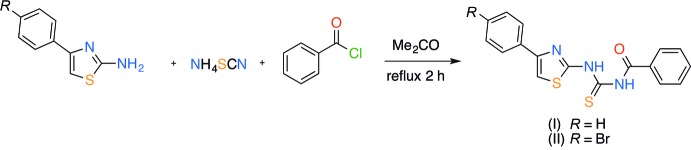
Synthesis of new thio­urea derivatives (I) and (II).

Benzoyl chloride (0.60 ml, 0.73 g, 5.19 mmol) was added over 5 min to a freshly prepared solution of NH_4_SCN (0.39 g, 5.19 mmol) in acetone (40 ml), and the mixture was heated under reflux for 15 min. After heating, the appropriate 4-aryl­thia­zol-2-amine (4.33 mmol) in acetone (10 ml) was added. The mixture was heated again under reflux for 2 h (Fig. 5 ▸). Then excess cracked ice was added with vigorous stirring. The resulting solid was collected and liberally washed with water. These compounds were isolated as pale-yellow crystalline solids in 41% and 45% yield for the 4-phenyl (I) and 4-(4-bromo­phen­yl) (II) derivatives, respectively. Single crystals of the products were obtained by slow crystallization from *N*,*N*-di­methyl­formamide solution.

**Spectroscopic and physical data for (I**): m.p. 481–483 K. FTIR ν_max_ cm^−1^: 3025, 1671, 1518, 1441, 1246, 1170, 668, 561. ^1^H NMR (600 MHz, DMSO-*d*_6_, 304 K): *δ* = 7.35 (*t*, 1H, *J* = 7.3), 7.45 (*t*, 2H, *J* = 7.6), 7.56 (*t*, 2H, *J* = 7.6), 7.69 (*t*, 1H, *J* = 7.4), 7.74 (*s*, 1H), 7.94 (*d*, 2H, *J* = 7.8), 8.03 (*d*, 2H, *J* = 7.8), 12.18 (*s*, 1H), 14.27 (*s*, 1H). Analysis calculated for C_17_H_13_N_3_OS_2_: C, 60.16; H, 3.86; N, 12.38. Found: C, 60.22; H, 3.93; N, 12.47.

**Spectroscopic and physical data for (II):** m.p. 484–486 K. FTIR ν_max_ cm^−1^: 3395, 3055, 1674, 1515, 1488, 1244, 1165, 697. ^1^H NMR (600 MHz, DMSO-*d*_6_,304 K): *δ* = 7.57 (*t*, 2H, *J* = 7.7), 7.64 (*d*, 2H, *J* = 8.0), 7.70 (*t*, 1H, *J* = 7.5), 7.83 (*s*, 1H), 7.90 (*d*, 2H, *J* = 8.1), 8.03 (*d*, 2H, *J* = 7.7), 1221 (*s*, 1H), 14.27 (*s*, 1H). Analysis calculated for C_17_H_12_N_3_OS_2_Br: C, 48.81; H, 2.89; N, 10.05. Found: C, 48.89; H, 2.95; N, 10.11.

## Refinement   

Crystal data, data collection and structure refinement details are summarized in Table 3 ▸. X-ray diffraction studies were carried out on the ‘Belok’ beamline (λ = 0.96990 Å) of the National Research Center ‘Kurchatov Institute’ (Moscow, Russian Federation) using a MAR CCD detector. For each compound, a total of 360 images were collected using an oscillation range of 1.0° (*φ* scan mode) and corrected for absorption using the *SCALA* program (Evans, 2006 ▸). The data were indexed, integrated and scaled using the utility *i*MOSFLM in the program *CCP4* (Battye *et al.*, 2011 ▸).

The hydrogen atoms of the amino groups were localized in the difference-Fourier map and included in the refinement with fixed positional (riding model) and isotropic displacement parameters [*U*_iso_(H) = 1.2*U*_eq_(N)]. The other hydrogen atoms were placed in calculated positions with C—H = 0.95 Å and refined using in a riding model with fixed isotropic displacement parameters [*U*_iso_(H) = 1.2*U*_eq_(C)].

## Supplementary Material

Crystal structure: contains datablock(s) global, I, II. DOI: 10.1107/S2056989016013396/hb7611sup1.cif

Structure factors: contains datablock(s) I. DOI: 10.1107/S2056989016013396/hb7611Isup2.hkl

Structure factors: contains datablock(s) II. DOI: 10.1107/S2056989016013396/hb7611IIsup3.hkl

Supporting information file. DOI: 10.1107/S2056989016013396/hb7611Isup4.cml

Supporting information file. DOI: 10.1107/S2056989016013396/hb7611IIsup5.cml

CCDC references: 1500238, 1500237

Additional supporting information:  crystallographic information; 3D view; checkCIF report

## Figures and Tables

**Table 1:** Hydrogen-bond geometry (Å, °) for (I)

*D*—H⋯*A*	*D*—H	H⋯*A*	*D*⋯*A*	*D*—H⋯*A*
N1—H1⋯O1	0.92	1.85	2.6145 (18)	139
N2—H2⋯S1^i^	0.93	2.69	3.5845 (15)	162
C13—H13⋯O1^ii^	0.95	2.44	3.299 (2)	150

Symmetry codes: (i) 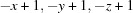; (ii) 
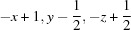
.

**Table 2:** Hydrogen-bond geometry (Å, °) for (II)

*D*—H⋯*A*	*D*—H	H⋯*A*	*D*⋯*A*	*D*—H⋯*A*
N1—H1⋯O1	0.88	1.93	2.644 (3)	138
N2—H2⋯Br1^i^	0.88	2.97	3.692 (3)	141
C13—H13⋯O1^ii^	0.95	2.53	3.340 (4)	144

Symmetry codes: (i) 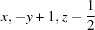; (ii) 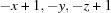.

**Table 3:** Experimental details

	(I)	(II)
Crystal data		
Chemical formula	C_17_H_13_N_3_OS_2_	C_17_H_12_BrN_3_OS_2_
*M*_r_	339.42	418.33
Crystal system, space group	Monoclinic, *P*2_1_/*c*	Monoclinic, *C*2/*c*
Temperature (K)	100	100
*a*, *b*, *c* (Å)	12.901 (3), 5.5160 (11), 23.143 (5)	37.210 (7), 4.0000 (8), 28.450 (6)
β (°)	105.32 (3)	128.69 (3)
*V* (Å^3^)	1588.4 (6)	3305.2 (18)
*Z*	4	8
Radiation type	Synchrotron, λ = 0.96990 Å	Synchrotron, λ = 0.96990 Å
μ (mm^−1^)	0.81	1.56
Crystal size (mm)	0.15 × 0.10 × 0.05	0.07 × 0.05 × 0.03

Data collection		
Diffractometer	MAR CCD	MAR CCD
Absorption correction	Multi-scan (*SCALA*; Evans, 2006 ▸)	Multi-scan (*SCALA*; Evans, 2006 ▸)
*T*_min_, *T*_max_	0.870, 0.950	0.880, 0.930
No. of measured, independent and observed [*I* > 2σ(*I*)] reflections	26393, 3395, 2899	13698, 3267, 2523
*R*_int_	0.033	0.065
(sin θ/λ)_max_ (Å^−1^)	0.642	0.641

Refinement		
*R*[*F* ^2^ > 2σ(*F* ^2^)], *wR*(*F* ^2^), *S*	0.036, 0.095, 1.03	0.040, 0.092, 1.02
No. of reflections	3395	3267
No. of parameters	209	217
H-atom treatment	H-atom parameters constrained	H-atom parameters constrained
Δρ_max_, Δρ_min_ (e Å^−3^)	0.32, −0.32	0.62, −0.78

Computer programs: *Automar* (MarXperts, 2015 ▸), *i* *MOSFLM* (Battye *et al.*, 2011 ▸), *SHELXS97* and *SHELXTL* (Sheldrick, 2008 ▸) and *SHELXL2014* (Sheldrick, 2015 ▸).

## supplementary crystallographic information

### (I) N-[(4-Phenylthiazol-2-yl)carbamothioyl]benzamide Crystal data

**Table:**

C_17_H_13_N_3_OS_2_	*F*(000) = 704
*M**_r_* = 339.42	*D*_x_ = 1.419 Mg m^−^^3^
Monoclinic, *P*2_1_/*c*	Synchrotron radiation, λ = 0.96990 Å
*a* = 12.901 (3) Å	Cell parameters from 600 reflections
*b* = 5.5160 (11) Å	θ = 2.4–34.0°
*c* = 23.143 (5) Å	µ = 0.81 mm^−^^1^
β = 105.32 (3)°	*T* = 100 K
*V* = 1588.4 (6) Å^3^	Prism, colourless
*Z* = 4	0.15 × 0.10 × 0.05 mm

### (I) N-[(4-Phenylthiazol-2-yl)carbamothioyl]benzamide Data collection

**Table:**

MAR CCD diffractometer	2899 reflections with *I* > 2σ(*I*)
φ scan	*R*_int_ = 0.033
Absorption correction: multi-scan (SCALA; Evans, 2006)	θ_max_ = 38.5°, θ_min_ = 2.2°
*T*_min_ = 0.870, *T*_max_ = 0.950	*h* = −16→16
26393 measured reflections	*k* = −6→7
3395 independent reflections	*l* = −29→28

### (I) N-[(4-Phenylthiazol-2-yl)carbamothioyl]benzamide Refinement

**Table:**

Refinement on *F*^2^	Secondary atom site location: difference Fourier map
Least-squares matrix: full	Hydrogen site location: mixed
*R*[*F*^2^ > 2σ(*F*^2^)] = 0.036	H-atom parameters constrained
*wR*(*F*^2^) = 0.095	*w* = 1/[σ^2^(*F*_o_^2^) + (0.0566*P*)^2^ + 0.566*P*] where *P* = (*F*_o_^2^ + 2*F*_c_^2^)/3
*S* = 1.03	(Δ/σ)_max_ = 0.001
3395 reflections	Δρ_max_ = 0.32 e Å^−^^3^
209 parameters	Δρ_min_ = −0.32 e Å^−^^3^
0 restraints	Extinction correction: SHELXL2014 (Sheldrick, 2015), Fc^*^=kFc[1+0.001xFc^2^λ^3^/sin(2θ)]^-1/4^
Primary atom site location: difference Fourier map	Extinction coefficient: 0.0035 (10)

### (I) N-[(4-Phenylthiazol-2-yl)carbamothioyl]benzamide Special details

**Table:**

Geometry. All esds (except the esd in the dihedral angle between two l.s. planes) are estimated using the full covariance matrix. The cell esds are taken into account individually in the estimation of esds in distances, angles and torsion angles; correlations between esds in cell parameters are only used when they are defined by crystal symmetry. An approximate (isotropic) treatment of cell esds is used for estimating esds involving l.s. planes.

### (I) N-[(4-Phenylthiazol-2-yl)carbamothioyl]benzamide Fractional atomic coordinates and isotropic or equivalent isotropic displacement parameters (Å^2^)

**Table:**

	*x*	*y*	*z*	*U*_iso_*/*U*_eq_	
S1	0.41550 (3)	0.80075 (7)	0.45047 (2)	0.03118 (14)	
S2	0.25885 (3)	1.09330 (7)	0.35070 (2)	0.02857 (14)	
O1	0.47826 (9)	0.3167 (2)	0.30362 (5)	0.0333 (3)	
N1	0.36884 (10)	0.6692 (2)	0.33491 (6)	0.0279 (3)	
H1	0.3853	0.5686	0.3068	0.033*	
N2	0.49953 (10)	0.4417 (2)	0.40077 (5)	0.0265 (3)	
H2	0.5346	0.4048	0.4403	0.032*	
N3	0.22879 (10)	0.8127 (2)	0.25771 (6)	0.0270 (3)	
C1	0.42612 (12)	0.6327 (3)	0.39203 (7)	0.0262 (3)	
C2	0.28765 (12)	0.8397 (3)	0.31264 (7)	0.0265 (3)	
C3	0.15407 (12)	0.9994 (3)	0.24243 (7)	0.0266 (3)	
C4	0.16011 (13)	1.1685 (3)	0.28659 (7)	0.0291 (3)	
H4	0.1159	1.3086	0.2826	0.035*	
C5	0.07557 (12)	0.9915 (3)	0.18273 (7)	0.0263 (3)	
C6	0.07731 (13)	0.7958 (3)	0.14375 (7)	0.0292 (3)	
H6	0.1300	0.6722	0.1555	0.035*	
C7	0.00219 (13)	0.7826 (3)	0.08808 (8)	0.0342 (4)	
H7	0.0038	0.6496	0.0623	0.041*	
C8	−0.07530 (13)	0.9630 (3)	0.06996 (8)	0.0356 (4)	
H8	−0.1264	0.9529	0.0320	0.043*	
C9	−0.07725 (14)	1.1582 (3)	0.10782 (8)	0.0343 (4)	
H9	−0.1298	1.2817	0.0956	0.041*	
C10	−0.00247 (13)	1.1736 (3)	0.16369 (7)	0.0303 (4)	
H10	−0.0043	1.3080	0.1891	0.036*	
C11	0.52613 (12)	0.3002 (3)	0.35705 (7)	0.0268 (3)	
C12	0.61647 (12)	0.1241 (3)	0.37854 (6)	0.0259 (3)	
C13	0.62162 (12)	−0.0727 (3)	0.34049 (7)	0.0280 (3)	
H13	0.5687	−0.0900	0.3034	0.034*	
C14	0.70420 (13)	−0.2414 (3)	0.35736 (7)	0.0298 (3)	
H14	0.7074	−0.3746	0.3319	0.036*	
C15	0.78246 (13)	−0.2151 (3)	0.41177 (7)	0.0318 (4)	
H15	0.8389	−0.3304	0.4232	0.038*	
C16	0.77780 (13)	−0.0197 (3)	0.44932 (7)	0.0316 (4)	
H16	0.8308	−0.0031	0.4864	0.038*	
C17	0.69563 (12)	0.1514 (3)	0.43264 (7)	0.0282 (3)	
H17	0.6934	0.2859	0.4579	0.034*	

### (I) N-[(4-Phenylthiazol-2-yl)carbamothioyl]benzamide Atomic displacement parameters (Å^2^)

**Table:**

	*U*^11^	*U*^22^	*U*^33^	*U*^12^	*U*^13^	*U*^23^
S1	0.0401 (2)	0.0298 (2)	0.0231 (2)	0.00420 (17)	0.00737 (16)	−0.00296 (15)
S2	0.0325 (2)	0.0253 (2)	0.0282 (2)	0.00129 (15)	0.00863 (15)	−0.00300 (15)
O1	0.0376 (6)	0.0382 (7)	0.0228 (6)	0.0089 (5)	0.0059 (5)	−0.0010 (5)
N1	0.0319 (7)	0.0282 (7)	0.0236 (6)	0.0033 (6)	0.0075 (5)	−0.0021 (5)
N2	0.0288 (6)	0.0286 (7)	0.0214 (6)	0.0022 (5)	0.0056 (5)	0.0004 (5)
N3	0.0287 (6)	0.0259 (7)	0.0270 (7)	0.0016 (5)	0.0086 (5)	0.0007 (5)
C1	0.0271 (7)	0.0260 (8)	0.0264 (7)	−0.0013 (6)	0.0087 (6)	0.0004 (6)
C2	0.0300 (8)	0.0232 (8)	0.0282 (8)	0.0003 (6)	0.0110 (6)	0.0005 (6)
C3	0.0276 (7)	0.0231 (8)	0.0314 (8)	0.0003 (6)	0.0120 (6)	0.0024 (6)
C4	0.0301 (8)	0.0259 (8)	0.0318 (8)	0.0019 (6)	0.0089 (6)	0.0003 (6)
C5	0.0265 (7)	0.0239 (8)	0.0300 (8)	−0.0012 (6)	0.0103 (6)	0.0026 (6)
C6	0.0272 (7)	0.0254 (8)	0.0342 (8)	0.0016 (6)	0.0066 (6)	−0.0001 (6)
C7	0.0342 (9)	0.0286 (9)	0.0375 (9)	0.0005 (7)	0.0058 (7)	−0.0038 (7)
C8	0.0300 (8)	0.0388 (10)	0.0340 (8)	0.0002 (7)	0.0017 (7)	0.0012 (7)
C9	0.0330 (8)	0.0323 (9)	0.0377 (9)	0.0087 (7)	0.0098 (7)	0.0063 (7)
C10	0.0336 (8)	0.0270 (8)	0.0328 (8)	0.0053 (7)	0.0131 (7)	0.0019 (6)
C11	0.0282 (8)	0.0290 (8)	0.0234 (7)	−0.0012 (6)	0.0074 (6)	0.0001 (6)
C12	0.0274 (7)	0.0271 (8)	0.0243 (7)	−0.0009 (6)	0.0091 (6)	0.0011 (6)
C13	0.0291 (8)	0.0306 (8)	0.0252 (7)	−0.0022 (6)	0.0085 (6)	−0.0013 (6)
C14	0.0336 (8)	0.0287 (8)	0.0301 (8)	0.0000 (7)	0.0135 (6)	−0.0015 (6)
C15	0.0320 (8)	0.0309 (9)	0.0343 (9)	0.0065 (7)	0.0120 (7)	0.0050 (7)
C16	0.0290 (8)	0.0387 (10)	0.0259 (8)	0.0022 (7)	0.0053 (6)	0.0020 (7)
C17	0.0317 (8)	0.0287 (8)	0.0250 (8)	−0.0014 (7)	0.0089 (6)	−0.0011 (6)

### (I) N-[(4-Phenylthiazol-2-yl)carbamothioyl]benzamide Geometric parameters (Å, º)

**Table:**

S1—C1	1.6747 (16)	C7—C8	1.394 (2)
S2—C4	1.7313 (17)	C7—H7	0.9500
S2—C2	1.7447 (16)	C8—C9	1.393 (3)
O1—C11	1.2308 (19)	C8—H8	0.9500
N1—C1	1.348 (2)	C9—C10	1.397 (2)
N1—C2	1.401 (2)	C9—H9	0.9500
N1—H1	0.9210	C10—H10	0.9500
N2—C11	1.3909 (19)	C11—C12	1.498 (2)
N2—C1	1.395 (2)	C12—C17	1.399 (2)
N2—H2	0.9300	C12—C13	1.410 (2)
N3—C2	1.306 (2)	C13—C14	1.391 (2)
N3—C3	1.391 (2)	C13—H13	0.9500
C3—C4	1.371 (2)	C14—C15	1.398 (2)
C3—C5	1.482 (2)	C14—H14	0.9500
C4—H4	0.9500	C15—C16	1.396 (2)
C5—C10	1.408 (2)	C15—H15	0.9500
C5—C6	1.411 (2)	C16—C17	1.395 (2)
C6—C7	1.395 (2)	C16—H16	0.9500
C6—H6	0.9500	C17—H17	0.9500
			
C4—S2—C2	88.06 (8)	C9—C8—H8	120.2
C1—N1—C2	128.58 (13)	C7—C8—H8	120.2
C1—N1—H1	115.7	C8—C9—C10	120.43 (15)
C2—N1—H1	115.7	C8—C9—H9	119.8
C11—N2—C1	127.36 (13)	C10—C9—H9	119.8
C11—N2—H2	116.3	C9—C10—C5	120.55 (15)
C1—N2—H2	116.3	C9—C10—H10	119.7
C2—N3—C3	110.42 (13)	C5—C10—H10	119.7
N1—C1—N2	115.46 (13)	O1—C11—N2	122.31 (14)
N1—C1—S1	124.67 (12)	O1—C11—C12	121.44 (14)
N2—C1—S1	119.87 (11)	N2—C11—C12	116.25 (13)
N3—C2—N1	117.88 (14)	C17—C12—C13	119.85 (14)
N3—C2—S2	115.87 (12)	C17—C12—C11	123.20 (14)
N1—C2—S2	126.22 (12)	C13—C12—C11	116.92 (13)
C4—C3—N3	114.53 (14)	C14—C13—C12	119.92 (14)
C4—C3—C5	127.25 (14)	C14—C13—H13	120.0
N3—C3—C5	118.17 (14)	C12—C13—H13	120.0
C3—C4—S2	111.10 (12)	C13—C14—C15	120.06 (15)
C3—C4—H4	124.5	C13—C14—H14	120.0
S2—C4—H4	124.5	C15—C14—H14	120.0
C10—C5—C6	118.47 (15)	C16—C15—C14	120.09 (15)
C10—C5—C3	121.79 (14)	C16—C15—H15	120.0
C6—C5—C3	119.73 (14)	C14—C15—H15	120.0
C7—C6—C5	120.38 (15)	C15—C16—C17	120.29 (15)
C7—C6—H6	119.8	C15—C16—H16	119.9
C5—C6—H6	119.8	C17—C16—H16	119.9
C8—C7—C6	120.58 (16)	C16—C17—C12	119.77 (15)
C8—C7—H7	119.7	C16—C17—H17	120.1
C6—C7—H7	119.7	C12—C17—H17	120.1
C9—C8—C7	119.56 (16)		
			
C2—N1—C1—N2	177.74 (14)	C5—C6—C7—C8	0.3 (3)
C2—N1—C1—S1	−3.2 (2)	C6—C7—C8—C9	0.2 (3)
C11—N2—C1—N1	5.9 (2)	C7—C8—C9—C10	−0.2 (3)
C11—N2—C1—S1	−173.23 (12)	C8—C9—C10—C5	−0.4 (2)
C3—N3—C2—N1	−178.70 (13)	C6—C5—C10—C9	0.9 (2)
C3—N3—C2—S2	−0.34 (17)	C3—C5—C10—C9	−178.30 (14)
C1—N1—C2—N3	−166.94 (15)	C1—N2—C11—O1	−6.7 (2)
C1—N1—C2—S2	14.9 (2)	C1—N2—C11—C12	173.58 (14)
C4—S2—C2—N3	−0.53 (12)	O1—C11—C12—C17	157.43 (15)
C4—S2—C2—N1	177.67 (14)	N2—C11—C12—C17	−22.9 (2)
C2—N3—C3—C4	1.36 (19)	O1—C11—C12—C13	−20.5 (2)
C2—N3—C3—C5	−176.47 (13)	N2—C11—C12—C13	159.15 (13)
N3—C3—C4—S2	−1.76 (17)	C17—C12—C13—C14	1.2 (2)
C5—C3—C4—S2	175.84 (12)	C11—C12—C13—C14	179.22 (13)
C2—S2—C4—C3	1.25 (12)	C12—C13—C14—C15	−0.5 (2)
C4—C3—C5—C10	2.7 (2)	C13—C14—C15—C16	0.1 (2)
N3—C3—C5—C10	−179.82 (13)	C14—C15—C16—C17	−0.5 (2)
C4—C3—C5—C6	−176.52 (15)	C15—C16—C17—C12	1.2 (2)
N3—C3—C5—C6	1.0 (2)	C13—C12—C17—C16	−1.6 (2)
C10—C5—C6—C7	−0.9 (2)	C11—C12—C17—C16	−179.47 (14)
C3—C5—C6—C7	178.33 (14)		

### (I) N-[(4-Phenylthiazol-2-yl)carbamothioyl]benzamide Hydrogen-bond geometry (Å, º)

**Table:**

*D*—H···*A*	*D*—H	H···*A*	*D*···*A*	*D*—H···*A*
N1—H1···O1	0.92	1.85	2.6145 (18)	139
N2—H2···S1^i^	0.93	2.69	3.5845 (15)	162
C13—H13···O1^ii^	0.95	2.44	3.299 (2)	150

Symmetry codes: (i) −*x*+1, −*y*+1, −*z*+1; (ii) −*x*+1, *y*−1/2, −*z*+1/2.

### (II) N-{[4-(4-Bromophenyl)thiazol-2-yl]carbamothioyl}benzamide Crystal data

**Table:**

C_17_H_12_BrN_3_OS_2_	*F*(000) = 1680
*M**_r_* = 418.33	*D*_x_ = 1.681 Mg m^−^^3^
Monoclinic, *C*2/*c*	Synchrotron radiation, λ = 0.96990 Å
*a* = 37.210 (7) Å	Cell parameters from 500 reflections
*b* = 4.0000 (8) Å	θ = 4.0–33.0°
*c* = 28.450 (6) Å	µ = 1.56 mm^−^^1^
β = 128.69 (3)°	*T* = 100 K
*V* = 3305.2 (18) Å^3^	Prism, colourless
*Z* = 8	0.07 × 0.05 × 0.03 mm

### (II) N-{[4-(4-Bromophenyl)thiazol-2-yl]carbamothioyl}benzamide Data collection

**Table:**

MAR CCD diffractometer	2523 reflections with *I* > 2σ(*I*)
φ scan	*R*_int_ = 0.065
Absorption correction: multi-scan (SCALA; Evans, 2006)	θ_max_ = 38.4°, θ_min_ = 4.0°
*T*_min_ = 0.880, *T*_max_ = 0.930	*h* = −44→44
13698 measured reflections	*k* = −4→4
3267 independent reflections	*l* = −32→32

### (II) N-{[4-(4-Bromophenyl)thiazol-2-yl]carbamothioyl}benzamide Refinement

**Table:**

Refinement on *F*^2^	Primary atom site location: difference Fourier map
Least-squares matrix: full	Secondary atom site location: difference Fourier map
*R*[*F*^2^ > 2σ(*F*^2^)] = 0.040	Hydrogen site location: inferred from neighbouring sites
*wR*(*F*^2^) = 0.092	H-atom parameters constrained
*S* = 1.02	*w* = 1/[σ^2^(*F*_o_^2^) + (0.02*P*)^2^] where *P* = (*F*_o_^2^ + 2*F*_c_^2^)/3
3267 reflections	(Δ/σ)_max_ = 0.002
217 parameters	Δρ_max_ = 0.62 e Å^−^^3^
0 restraints	Δρ_min_ = −0.78 e Å^−^^3^

### (II) N-{[4-(4-Bromophenyl)thiazol-2-yl]carbamothioyl}benzamide Special details

**Table:**

Geometry. All esds (except the esd in the dihedral angle between two l.s. planes) are estimated using the full covariance matrix. The cell esds are taken into account individually in the estimation of esds in distances, angles and torsion angles; correlations between esds in cell parameters are only used when they are defined by crystal symmetry. An approximate (isotropic) treatment of cell esds is used for estimating esds involving l.s. planes.

### (II) N-{[4-(4-Bromophenyl)thiazol-2-yl]carbamothioyl}benzamide Fractional atomic coordinates and isotropic or equivalent isotropic displacement parameters (Å^2^)

**Table:**

	*x*	*y*	*z*	*U*_iso_*/*U*_eq_	
Br1	0.66455 (2)	0.24056 (7)	0.96731 (2)	0.02282 (14)	
S1	0.67189 (2)	0.95707 (16)	0.58750 (3)	0.02136 (19)	
S2	0.70200 (2)	0.97423 (15)	0.71326 (3)	0.01827 (18)	
O1	0.55011 (6)	0.3045 (5)	0.53088 (9)	0.0239 (5)	
N1	0.62216 (7)	0.6652 (5)	0.61537 (10)	0.0176 (5)	
H1	0.5969	0.5551	0.6017	0.021*	
N2	0.59498 (7)	0.6073 (5)	0.51692 (9)	0.0194 (5)	
H2	0.5969	0.6736	0.4890	0.023*	
N3	0.64079 (7)	0.6390 (5)	0.71031 (9)	0.0178 (5)	
C1	0.62849 (9)	0.7359 (6)	0.57480 (13)	0.0183 (7)	
C2	0.65111 (9)	0.7460 (6)	0.67684 (13)	0.0173 (6)	
C3	0.67476 (9)	0.7355 (6)	0.76996 (13)	0.0167 (6)	
C4	0.70985 (8)	0.9180 (6)	0.77919 (12)	0.0197 (6)	
H4	0.7353	1.0029	0.8172	0.024*	
C5	0.67149 (8)	0.6265 (6)	0.81672 (11)	0.0175 (6)	
C6	0.71033 (9)	0.6346 (7)	0.87798 (12)	0.0213 (6)	
H6	0.7387	0.7176	0.8894	0.026*	
C7	0.70779 (8)	0.5233 (6)	0.92191 (12)	0.0219 (7)	
H7	0.7343	0.5286	0.9631	0.026*	
C8	0.66619 (8)	0.4035 (6)	0.90546 (12)	0.0193 (6)	
C9	0.62698 (9)	0.3949 (7)	0.84512 (12)	0.0223 (6)	
H9	0.5986	0.3141	0.8340	0.027*	
C10	0.63006 (9)	0.5062 (6)	0.80154 (12)	0.0216 (6)	
H10	0.6034	0.5006	0.7604	0.026*	
C11	0.55924 (8)	0.3889 (6)	0.49790 (12)	0.0187 (6)	
C12	0.53267 (9)	0.2627 (6)	0.43455 (13)	0.0184 (7)	
C13	0.48960 (8)	0.1152 (7)	0.40853 (12)	0.0216 (6)	
H13	0.4785	0.1002	0.4308	0.026*	
C14	0.46331 (9)	−0.0089 (6)	0.34991 (12)	0.0239 (7)	
H14	0.4341	−0.1062	0.3321	0.029*	
C15	0.47950 (9)	0.0089 (6)	0.31750 (13)	0.0253 (7)	
H15	0.4613	−0.0754	0.2775	0.030*	
C16	0.52255 (9)	0.1505 (7)	0.34332 (13)	0.0262 (7)	
H16	0.5337	0.1612	0.3210	0.031*	
C17	0.54912 (9)	0.2757 (6)	0.40176 (13)	0.0225 (7)	
H17	0.5785	0.3702	0.4194	0.027*	

### (II) N-{[4-(4-Bromophenyl)thiazol-2-yl]carbamothioyl}benzamide Atomic displacement parameters (Å^2^)

**Table:**

	*U*^11^	*U*^22^	*U*^33^	*U*^12^	*U*^13^	*U*^23^
Br1	0.0213 (2)	0.0297 (2)	0.0189 (2)	0.00396 (11)	0.01329 (17)	0.00383 (11)
S1	0.0214 (3)	0.0250 (3)	0.0184 (4)	−0.0048 (3)	0.0128 (3)	−0.0023 (3)
S2	0.0148 (3)	0.0215 (3)	0.0161 (4)	−0.0005 (2)	0.0085 (3)	−0.0005 (2)
O1	0.0196 (10)	0.0343 (11)	0.0182 (11)	−0.0039 (8)	0.0119 (9)	0.0011 (8)
N1	0.0130 (10)	0.0241 (11)	0.0136 (12)	−0.0010 (8)	0.0073 (10)	0.0011 (9)
N2	0.0181 (10)	0.0249 (12)	0.0119 (11)	−0.0010 (9)	0.0078 (9)	0.0018 (9)
N3	0.0145 (10)	0.0206 (11)	0.0136 (11)	0.0014 (9)	0.0066 (9)	0.0006 (9)
C1	0.0154 (13)	0.0199 (13)	0.0149 (15)	0.0050 (9)	0.0072 (12)	0.0032 (9)
C2	0.0154 (13)	0.0217 (14)	0.0132 (14)	0.0026 (9)	0.0082 (12)	0.0013 (9)
C3	0.0144 (13)	0.0169 (13)	0.0156 (15)	0.0024 (9)	0.0079 (12)	−0.0009 (9)
C4	0.0179 (12)	0.0214 (13)	0.0144 (14)	0.0000 (10)	0.0075 (11)	−0.0006 (10)
C5	0.0171 (12)	0.0196 (12)	0.0132 (13)	0.0035 (10)	0.0083 (11)	0.0004 (10)
C6	0.0141 (12)	0.0292 (14)	0.0185 (15)	0.0000 (11)	0.0091 (12)	0.0000 (11)
C7	0.0141 (12)	0.0308 (15)	0.0129 (14)	0.0024 (10)	0.0046 (11)	−0.0020 (10)
C8	0.0187 (12)	0.0231 (13)	0.0160 (14)	0.0038 (10)	0.0108 (11)	0.0004 (10)
C9	0.0186 (13)	0.0268 (14)	0.0211 (15)	−0.0028 (11)	0.0122 (12)	−0.0021 (11)
C10	0.0169 (12)	0.0291 (14)	0.0146 (14)	0.0000 (10)	0.0078 (11)	−0.0017 (10)
C11	0.0131 (12)	0.0221 (13)	0.0142 (14)	0.0019 (10)	0.0054 (11)	0.0031 (10)
C12	0.0135 (13)	0.0212 (14)	0.0144 (15)	0.0023 (9)	0.0057 (12)	0.0015 (9)
C13	0.0174 (13)	0.0244 (14)	0.0193 (15)	0.0022 (11)	0.0097 (12)	0.0018 (11)
C14	0.0148 (13)	0.0256 (14)	0.0218 (15)	−0.0017 (10)	0.0068 (12)	−0.0014 (11)
C15	0.0212 (13)	0.0269 (15)	0.0161 (14)	0.0000 (10)	0.0059 (12)	−0.0025 (11)
C16	0.0268 (15)	0.0312 (15)	0.0199 (15)	−0.0015 (12)	0.0142 (13)	−0.0025 (12)
C17	0.0153 (13)	0.0269 (15)	0.0186 (16)	−0.0029 (10)	0.0073 (13)	−0.0004 (10)

### (II) N-{[4-(4-Bromophenyl)thiazol-2-yl]carbamothioyl}benzamide Geometric parameters (Å, º)

**Table:**

Br1—C8	1.913 (3)	C6—H6	0.9500
S1—C1	1.670 (3)	C7—C8	1.393 (4)
S2—C4	1.723 (3)	C7—H7	0.9500
S2—C2	1.743 (3)	C8—C9	1.395 (4)
O1—C11	1.228 (4)	C9—C10	1.388 (4)
N1—C1	1.345 (4)	C9—H9	0.9500
N1—C2	1.403 (4)	C10—H10	0.9500
N1—H1	0.8800	C11—C12	1.501 (4)
N2—C11	1.387 (3)	C12—C17	1.401 (5)
N2—C1	1.401 (3)	C12—C13	1.408 (4)
N2—H2	0.8800	C13—C14	1.394 (4)
N3—C2	1.302 (4)	C13—H13	0.9500
N3—C3	1.394 (3)	C14—C15	1.383 (4)
C3—C4	1.370 (4)	C14—H14	0.9500
C3—C5	1.476 (4)	C15—C16	1.398 (4)
C4—H4	0.9500	C15—H15	0.9500
C5—C10	1.401 (4)	C16—C17	1.392 (4)
C5—C6	1.407 (3)	C16—H16	0.9500
C6—C7	1.385 (4)	C17—H17	0.9500
			
C4—S2—C2	87.75 (14)	C7—C8—Br1	118.4 (2)
C1—N1—C2	127.7 (2)	C9—C8—Br1	120.9 (2)
C1—N1—H1	116.1	C10—C9—C8	119.0 (3)
C2—N1—H1	116.1	C10—C9—H9	120.5
C11—N2—C1	128.4 (3)	C8—C9—H9	120.5
C11—N2—H2	115.8	C9—C10—C5	121.6 (2)
C1—N2—H2	115.8	C9—C10—H10	119.2
C2—N3—C3	110.0 (2)	C5—C10—H10	119.2
N1—C1—N2	115.3 (2)	O1—C11—N2	122.0 (3)
N1—C1—S1	126.2 (2)	O1—C11—C12	122.4 (2)
N2—C1—S1	118.6 (2)	N2—C11—C12	115.6 (3)
N3—C2—N1	119.2 (2)	C17—C12—C13	119.6 (3)
N3—C2—S2	116.4 (2)	C17—C12—C11	123.6 (2)
N1—C2—S2	124.3 (2)	C13—C12—C11	116.8 (3)
C4—C3—N3	114.3 (3)	C14—C13—C12	119.7 (3)
C4—C3—C5	126.2 (2)	C14—C13—H13	120.2
N3—C3—C5	119.5 (2)	C12—C13—H13	120.2
C3—C4—S2	111.6 (2)	C15—C14—C13	120.4 (3)
C3—C4—H4	124.2	C15—C14—H14	119.8
S2—C4—H4	124.2	C13—C14—H14	119.8
C10—C5—C6	118.1 (3)	C14—C15—C16	120.3 (3)
C10—C5—C3	121.2 (2)	C14—C15—H15	119.9
C6—C5—C3	120.7 (2)	C16—C15—H15	119.9
C7—C6—C5	120.9 (3)	C17—C16—C15	119.9 (3)
C7—C6—H6	119.5	C17—C16—H16	120.1
C5—C6—H6	119.5	C15—C16—H16	120.1
C6—C7—C8	119.7 (2)	C16—C17—C12	120.2 (3)
C6—C7—H7	120.1	C16—C17—H17	119.9
C8—C7—H7	120.1	C12—C17—H17	119.9
C7—C8—C9	120.7 (3)		
			
C2—N1—C1—N2	176.6 (2)	C6—C7—C8—C9	−0.1 (4)
C2—N1—C1—S1	−3.2 (4)	C6—C7—C8—Br1	178.3 (2)
C11—N2—C1—N1	−7.5 (4)	C7—C8—C9—C10	0.3 (4)
C11—N2—C1—S1	172.2 (2)	Br1—C8—C9—C10	−178.0 (2)
C3—N3—C2—N1	177.7 (2)	C8—C9—C10—C5	0.0 (4)
C3—N3—C2—S2	−0.5 (3)	C6—C5—C10—C9	−0.5 (4)
C1—N1—C2—N3	−175.8 (2)	C3—C5—C10—C9	178.3 (2)
C1—N1—C2—S2	2.2 (4)	C1—N2—C11—O1	7.4 (4)
C4—S2—C2—N3	0.2 (2)	C1—N2—C11—C12	−172.4 (2)
C4—S2—C2—N1	−177.9 (2)	O1—C11—C12—C17	−161.6 (2)
C2—N3—C3—C4	0.6 (3)	N2—C11—C12—C17	18.2 (3)
C2—N3—C3—C5	−176.9 (2)	O1—C11—C12—C13	16.4 (4)
N3—C3—C4—S2	−0.5 (3)	N2—C11—C12—C13	−163.8 (2)
C5—C3—C4—S2	176.8 (2)	C17—C12—C13—C14	−1.6 (4)
C2—S2—C4—C3	0.17 (19)	C11—C12—C13—C14	−179.6 (2)
C4—C3—C5—C10	165.8 (2)	C12—C13—C14—C15	0.7 (4)
N3—C3—C5—C10	−17.0 (4)	C13—C14—C15—C16	0.3 (4)
C4—C3—C5—C6	−15.5 (4)	C14—C15—C16—C17	−0.4 (4)
N3—C3—C5—C6	161.7 (2)	C15—C16—C17—C12	−0.5 (4)
C10—C5—C6—C7	0.7 (4)	C13—C12—C17—C16	1.5 (4)
C3—C5—C6—C7	−178.1 (2)	C11—C12—C17—C16	179.4 (2)
C5—C6—C7—C8	−0.4 (4)		

### (II) N-{[4-(4-Bromophenyl)thiazol-2-yl]carbamothioyl}benzamide Hydrogen-bond geometry (Å, º)

**Table:**

*D*—H···*A*	*D*—H	H···*A*	*D*···*A*	*D*—H···*A*
N1—H1···O1	0.88	1.93	2.644 (3)	138
N2—H2···Br1^i^	0.88	2.97	3.692 (3)	141
C13—H13···O1^ii^	0.95	2.53	3.340 (4)	144

Symmetry codes: (i) *x*, −*y*+1, *z*−1/2; (ii) −*x*+1, −*y*, −*z*+1.
